# Evaluating the role of dietary interventions in reducing chemotherapy toxicities in cancer patients: a systematic review

**DOI:** 10.1007/s11764-025-01777-6

**Published:** 2025-03-22

**Authors:** Jessica Abene, Jie Deng

**Affiliations:** School of Nursing, University of Pennsylvania, 418 Curie Blvd, Philadelphia, PA USA

**Keywords:** Chemotherapy toxicities, Quality of life, Diet, Malignancy, Cancer therapy, Chemotherapy

## Abstract

**Purpose:**

This systematic review synthesizes current literature examining the relationship between various dietary patterns and chemotherapy toxicities among patients currently receiving chemotherapy treatment. It aims to determine the most advantageous dietary pattern for patients with various malignancies.

**Methods:**

PubMed, CINAHL, and Embase were thoroughly searched to select quantitative studies that answered the research question and met the inclusion criteria as of July 2024.

**Results:**

Fourteen studies were analyzed: 10 randomized control trials (RCTs), 2 quasi-experimental, 1 case series, and 1 cohort study. Analyses were stratified by dietary pattern: fasting; Mediterranean diet; ketogenic diet; plant-based, high-protein diet; and anti-inflammatory diet. The results suggest that these all have the ability to benefit patients’ experiences with chemotherapy. Fasting; a Mediterranean diet; a plant-based, high-protein diet; and an anti-inflammatory diet may reduce toxicities such as fatigue, diarrhea, insomnia, and nausea throughout chemotherapy. The ketogenic diet might offer initial advantages in improving diarrhea and physical activity; however, these results may not be sustainable.

**Conclusions:**

There is insufficient evidence to identify a single dietary intervention as the most effective in reducing chemotherapy toxicities in patients undergoing chemotherapy. Further research is necessary to uncover the most beneficial dietary pattern to recommend as a nutritional strategy for survivors undergoing chemotherapy.

**Implications for Cancer Survivors:**

Survivors may benefit from adopting specific dietary patterns to alleviate chemotherapy toxicities. These reductions may improve chemotherapy tolerance, minimize treatment interruptions, and enhance daily functioning.

**Supplementary Information:**

The online version contains supplementary material available at https://doi.org/10.1007/s11764-025-01777-6.

## Introduction

According to the World Health Organization (WHO), approximately 1 in 5 people will be diagnosed with cancer during their lifetime, maintaining cancer’s spot as one of the leading causes of death globally [1]. It is estimated that by 2050, there will be 35 million new cancer diagnoses. This is a 77% increase in cancer cases from the year 2022 [1]. Despite advancements in cancer treatment, chemotherapy remains an indispensable treatment modality. The National Cancer Institute defines toxicity as “the extent to which something is poisonous or harmful” [2]. This paper uses the term “toxicity” to refer to the harmful effects that chemotherapy drugs have on normal, healthy cells due to their toxic composition, resulting in unpleasant effects such as nausea, fatigue, and organ damage [3, 4]. These toxicities can vary in severity and may significantly impact a patient’s quality of life (QOL) during treatment.

Studies have shown that 97.4% of patients receiving chemotherapy face at least one toxicity and approximately three-quarters will experience multiple toxicities from their regimen such as fatigue, loss of appetite, and diarrhea [3, 4]. Moreover, 66.7% of patients receiving chemotherapy experience six or more toxicities [4]. Toxicities often continuously interfere with patients’ lives for months after their chemotherapy treatment has ended [4, 5]. Frequently, it is communicated that these toxicities are more detrimental to patients’ QOL than the malignancy itself [3, 6]. Many cancers are also treated with several chemotherapy drugs, and individuals receiving multi-drug therapy often experience higher rates of toxicity, further deteriorating QOL [7]. Multiple studies have shown that QOL is a predictor of survival in various malignancies, making it an important aspect to focus on during cancer treatment [3, 8].

Unfortunately, patients receiving chemotherapy continue to struggle with toxicities despite pharmacological intervention, making it clear that medication alone is not adequate to manage these unpleasant toxicities [9, 10]. For example, 44% of patients receiving chemotherapy experience breakthrough nausea and vomiting despite antiemetic prophylaxis, highlighting that medication-based treatments still leave a significant proportion of patients dealing with toxicities [9]. A more novel pathway that is frequently overlooked for possibly mitigating the toxicities of chemotherapy is dietary intervention. That diet is an important aspect of human health is well established. Epidemiology has shown a strong association between obesity and cancer; therefore, it is known that maintaining a healthy diet may reduce an individual’s risk of developing cancer and the overall prevalence of the disease [11–13]. Less is understood about the influence of diet on cancer after diagnosis, specifically with regard to its impact on cancer treatment. To date, the only broadly accepted nutritional interventions for patients with malignancies involve the management of nutrition impact symptoms (e.g., mouth sores), cancer-related malnutrition, and obesity [11, 14]. Dietary patterns such as ketogenic; Mediterranean; plant-based, high-protein; anti-inflammatory; and various forms of fasting are among the current research for having the potential to improve chemotherapy-related toxicities.

While there is limited literature examining the relationship between diet and chemotherapy, there is currently no comprehensive review that compares the influence different dietary patterns have on patients’ experiences with chemotherapy. A systematic review was conducted to investigate the impact of various dietary patterns on chemotherapy toxicities in patients with cancer. The purpose of this review is to (1) identify literature that investigates dietary patterns’ impact on chemotherapy toxicities in patients currently receiving treatment; (2) evaluate the methodological rigor of the studies identified; (3) compare and contrast the results of these studies; (4) recognize the dietary pattern that appears to be the most advantageous for patients undergoing chemotherapy; and (5) suggest future research avenues in this field.

## Information sources and methods

The Preferred Reporting Items for Systematic Reviews and Meta-Analyses (PRISMA) guidelines guided the systematic approach of this paper. A comprehensive literature search was conducted in July 2024. A health sciences University Librarian assisted in selecting databases and developing a search strategy. The following databases were searched: PubMed, Cumulated Index to Nursing and Allied Health Literature (CINAHL), and Embase. Search terms (Appendix) were chosen by reviewing related literature, and synonymous terms were sourced from the Medical Subject Headings (MeSH) database. The searches were filtered for quantitative literature written in English and published since 2008.

### Study selection and screening process

Articles were screened at the title/abstract and full-text level for eligibility based on the inclusion and exclusion criteria. To be included in the review, studies had to: (1) be a quantitative study; (2) investigate a dietary pattern’s impact on chemotherapy toxicities; and (3) include human participants (≥ 18 years old) currently on chemotherapy treatment. Studies were not limited by the type of malignancy. Studies excluded were those that: (1) were not published in English; (2) examine cancer therapy besides chemotherapy (e.g., radiation); (3) include animal subjects; (4) include human participants during post-treatment or survivorship; (5) investigate diet’s role in cancer prevention; (6) have multi-modality interventions (e.g., diet and exercise); and (7) focus on elemental or supplemental diets (e.g., pre-digested nutrition formulations and single-nutrient supplements).

**Fig. 1:**
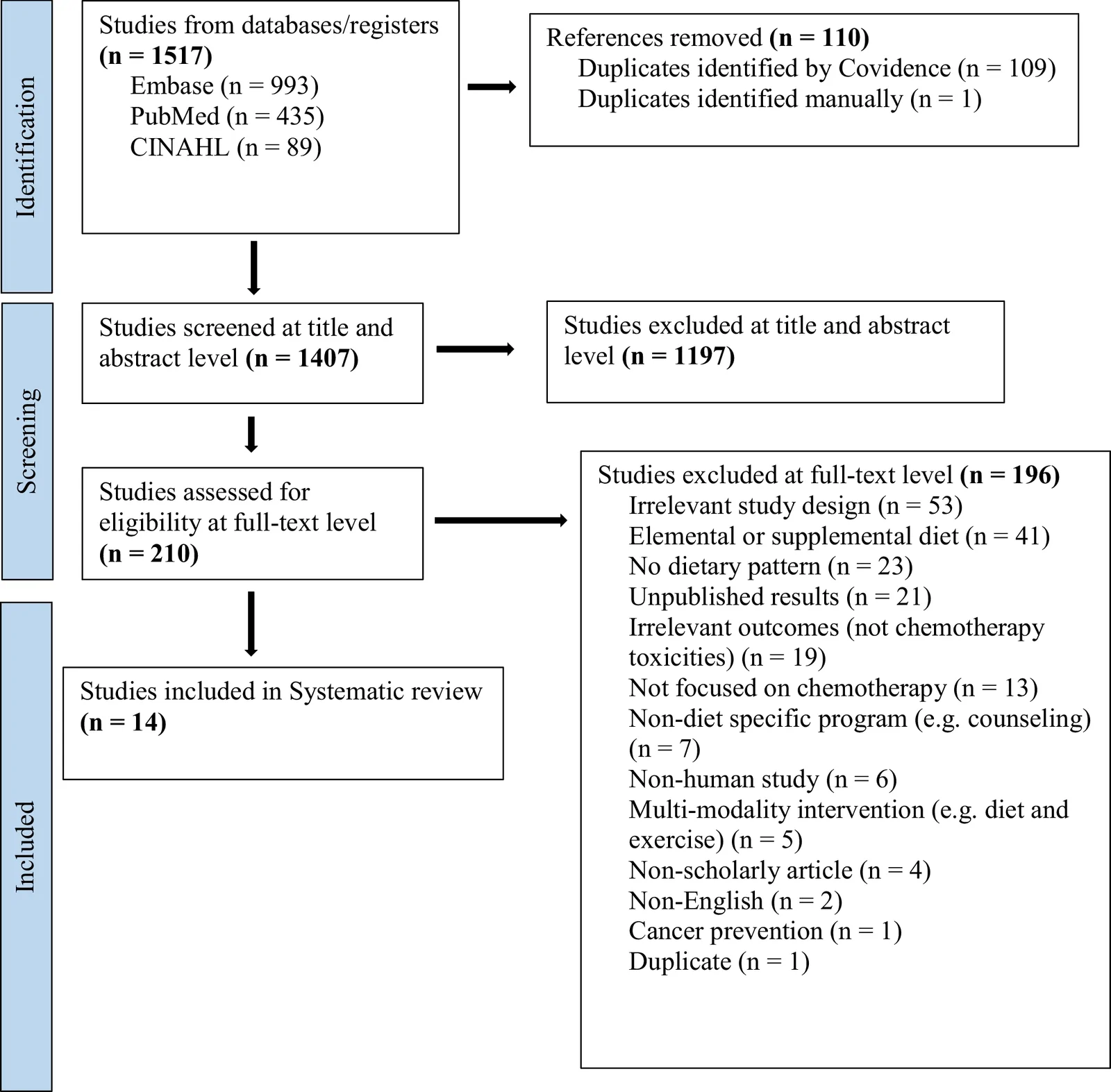
PRISMA flow diagram for the selection of studies

The systematic review management tool, Covidence, was used to organize the articles extracted and remove duplicates. The search in July 2024 resulted in a total of 1517 records. After the removal of 110 duplicates and the title and abstract screen, 210 articles were assessed at the full-text level. Fourteen studies were considered suitable for the objectives of this review and were included in the analysis (Fig. 1 PRISMA). The primary reasons for exclusion include irrelevant study design, use of elemental or supplemental dietary interventions, absence of dietary pattern, unpublished results, irrelevant outcomes, and lack of focus on chemotherapy.

### Quality assessment

A critical appraisal for potential risks of bias was performed for each study presented in this review. The Joanna Briggs Institute’s (JBI) critical appraisal tools were selected for their tailored instruments designed specifically for quantitative studies, offering clear, checklist-based questions to effectively assess the risk of bias [15]. The tools for cohort, case studies, quasi-experimental, and randomized control trials (RCTs) were applied. Separate tables depicting the quality assessment for each study design are included (Supplementary Tables 1–4). The responses to the items in the tool were recorded as yes (1), no/unclear (0), or not applicable (X). Each study was rated on a categorical scale (poor, fair, good, and excellent), with the rating determined by the percentage of 'yes' responses out of the total items in the tool: poor (0–25%), fair (26–50%), good (51–75%), and excellent (76–100%).

### Data extraction

Data extracted included study design, study characteristics (setting, sample size (*n*), sex, type and stage of malignancy, chemotherapy regimen, and study limitations), intervention and control, study duration, outcome measures, and summary of results (Tables 1, 2). An Excel spreadsheet was utilized to organize the data and create the official study tables included in this review. The checklists for JBI’s critical appraisal tools were downloaded as Word documents and filled out individually for each article.

**Table 1:** Characteristics of studies

Authors	Study design	Level of evidence	Setting	Study size (n)	Sex (F/M)	Malignancy	Tumor stage (I–IV)	Chemotherapy regimen	Limitations	Quality assessment rating (poor, fair, good, excellent)
Alshammari et al., 2023	Cohort study	Level 5	Riyadh, Saudi Arabia	37	54.1% F, 45.9% M	100% colorectal	5.4% II, 27.0% III, 67.6% IV	5-fluorouracil-base chemotherapies (FOLFOX or FOLFIRI regimen: includes fluorouracil and leucovorin added to either oxaliplatin or irinotecan)	Small sample size, the majority of participants having stage IV cancer could have overestimated CEA and/or LDH levels	Good
Artale et al., 2022	Quasi-experimental	Level 3	Edinburgh, Scottland	137	54% F, 46% M	100% colorectal	Metastatic	Chemotherapy with fluoropyrimidine, oxaliplatin, and irinotecan (irinotecan alone, CAPOX, FOLFOX, or FOLFIRI regimens)	No control arm, subjects from a single clinical center, behavioral and environmental changes possible due to the pandemic outbreak extending programmed length	Fair
Bauersfeld et al., 2018	RCT cross-over	Level 2	Berlin, Germany	50	100% F	88.2% breast, 11.7% ovarian	Not reported	Taxanes (docetaxel (D), paclitaxel (T)), platinum agents (carboplatin (P), cyclophosphamide (C)), anthracyclines (epirubicin (E), doxorubicin (A), methotrexate (M), fluorouracil (F)), IgG1 antibody bevacizumab (Avastin), and for HER2/neu overexpression pertuzumab or trastuzumab; breast cancer regimens: EC, FEC-D; FEC-D + trastuzumab; AC-T, EC-T, TAC; D + pertuzumab + trastuzumab; ovarian cancer regimens: P mono, P + T, EC-T + P, P + T + bevacizumab	Small sample size; heterogeneity in the study population and treatment; underreporting and non-compliance issues surrounding fasting; cross-over design could have led to carry-over effects; the positive reputation of fasting in Germany could have led to a non-specific effect; the group who implemented fasting at the start of chemotherapy had a lower baseline QOL score than participants who did not begin fasting towards the middle of treatment, which may explain their better response to the intervention	Good
de Groot et al., 2020	RCT	Level 2	Netherlands	131	100% F	100% HER2- negative breast	1.6% I, 76.7% II, 22.5% III	76.7% neo-adjuvant AC-T, 23.3% neo-adjuvant FEC-T	Risk for self-selection bias: some patients in the control group decided to fast on their own initiative which may have diminished the positive impact of the FMD	Excellent
de Groot et al., 2015	RCT	Level 2	Netherlands	13	100% F	100% HER2- negative breast	69.2% II, 30.8% III	61.5% adjuvant and 38.5% neo-adjuvant TAC (docetaxel, doxorubicin, cyclophosphamide) with granulocyte-colony stimulating factor	Small sample size, relatively short fasting period (48 h), the high dose of dexamethasone given and the large variability of toxicities between patients may have masked the effects of STF, protocol may not have been rapid enough to pick up on DNA damage since it is often repaired quickly	Good
Khodabakhshi et al., 2020	RCT	Level 2	Tehran, Iran	80	100% F	100% breast	73.3% locally advanced disease, 26.7% metastatic disease	Not reported	Small sample size, heterogeneous sample in terms of cancer stage	Good
Kleckner et al., 2022	RCT	Level 2	USA	33	93.9% F, 6.1% M	90.9% breast, 9.1% other	24.2% I, 63.6% II, 6.1% III, 6.1% other or unknown	33.3% doxorubicin cyclophosphamide (AC), 21.3% paclitaxel (with or without trastuzumab), 21.2% docetaxel carboplatin trastuzumab pertuzumab (TCHP), 12.1% docetaxel cyclophosphamide (TC), 12.1% other non-anthracyclines	Sample was not generalizable given participants were mostly highly-educated, non-Hispanic White women with breast cancer; the three data collection points for fatigue may not have captured the true trajectory of cancer-related fatigue; blood samples were not collected fasted which may have caused increased variability; the pandemic may have impacted diet adherence; participants and analysis were not blinded; control was not a time- and attention- intervention	Good
Lugtenberg et al., 2021	RCT	Level 2	Netherlands	131	100% F	100% HER2- negative breast	1.6% I, 76.7% II, 22.5% III	76.7% neo-adjuvant AC-T, 23.3% neo-adjuvant FEC-T	Possibility of bias because questionnaire completion declined over the trial, questionnaire completion rates differed between groups, those who felt better throughout the trail may have been more likely to follow the intervention, lack of blinding could have impacted patients' perceived outcomes	Excellent
Omar et al., 2022	RCT	Level 2	Damanhour, Egypt	48	Not reported	100% HER2- negative breast	20.8% IA, 35.4% IIA, 12.5% IIB, 10.4% IIIA, 20.8% IIIC	100% adjuvant AC (doxorubicin, cyclophosphamide)	Possibility of incomplete adherence to the fasting hours and allotted consumption of food	Excellent
Riedinger et al., 2020	RCT	Level 2	USA	24	100% F	55% ovarian, 40% uterine, 5% cervical	15% I, 5% II, 65% III, 15% IV	90% platinum + taxane, 5% doxorubicin, 5% gemcitabine + taxane; 16.7% being neo-adjuvant chemotherapy	Small sample size, homogeneity of patient demographics, relied on patient-reported compliance rather than confirming compliance through laboratory tests	Good
Safdie et al., 2009	Case series	Level 6	USA	10	70% F, 30% M	40% breast, 20% prostate, 10% ovarian, 10% uterine, 10% NSCLC, 10% esophageal adenocarcinoma	10% IA, 10% II, 40% IIA, 20% IV, 10% IVB, 10% IV/DI	30% docetaxel + cyclophosphamide, 20% docetaxel, 20% docetaxel + carboplatin, 10% docetaxel + carboplatin + 5-fluorouracil, 10% carboplatin + paclitaxel, 10% gemcitabine + gemcitabine docetaxel; 50% being adjuvant chemotherapy	Small sample size, bias could be present in the self-report of side effects	Good
Sathiaraj et al., 2023	RCT	Level 2	Bengaluru, India	103	100% F	100% breast	9.7% I, 49.5% II, 40.7% III	Adjuvant doxorubicin hydrochloride (adriamycin) + cyclophosphamide (AC) or adjuvant docetaxel (taxotere) + cyclophosphamide (TC)	Small sample size, blinding not possible except for those who analyzed data, not a multicenter trial, accuracy of dietary recall cannot be certain	Good
Zhang et al., 2023	RCT	Level 2	China	106	26.5% F, 73.5% M	Lung: 51% adenocarcinoma, 28.6% squamous cell carcinoma, 20.4% small cell/other	13.3% I-II, 86.7% III-IV	Not reported	Self-report questionnaires may not be accurate, body composition was not measured, only one inflammatory marker was tested (hs-CPR)	Excellent
Zorn et al., 2020	Quasi-experimental cross-over	Level 3	Germany	51	100% F	73.3% breast, 13.3% cervical, 6.7% endometrial, 6.7% ovarian	I-IV (no percentage reported)	73.3% epirubicin/cyclophosphamid, 23.3% paclitaxel/carboplatin, 3.3% docetaxel/cyclophosphamid; 73.3% neo-adjuvant, 20% adjuvant, 10% palliative chemotherapy	Small sample size, no randomization, groups were of unequal size, patients' characteristics differed significantly between the groups, high dropout rate, failed to document concomitant medication	Excellent

*CEA*, carcinoembryonic antigen; *LDH*, lactate dehydrogenase; *RCT*, randomized control trial; *HER2*, human epidermal growth factor receptor 2; *AC-T*, doxorubicin/cyclophosphamide followed by docetaxel; *FEC-T*, fluorouracil/epirubicin/cyclophosphamide; *FMD*, fasting mimicking diet; *STF*, short-term fasting; *DNA*, deoxyribonucleic acid; *USA*, United States of America; *NSCLC*, non-small cell lung cancer; *hs-CPR*, high-sensitivity C-reactive protein

**Table 2:** Summary of study results

Study	Dietary intervention	Dietary comparison	Study duration	Chemotherapy toxicity outcome measures	Results	Conclusions
Alshammari et al., 2023	IF: refrain from eating and drinking (including oral medications) from sunrise to sunset; Group A (10.8% did not fast, 18.9% fasted < 20 days), Group B (35.1% fasted the entire 30 days of Ramadan, 35.1% fasted ≥ 20 days)	NA	Throughout the celebration of Ramadan: 49 days	Questionnaire-based survey	73% of patients reported improved tolerability of chemotherapy side effects; although nausea only worsened in less than 20% of patients, it did not improve QOL	IF may enhance chemotherapy tolerability
Artale et al., 2022	MMHD: small and frequent meals at room temperature and containing mainly dry foods; daily caloric intake of 1818 kcal; reduced fiber intake (22.90–30.36 g); limited legumes, vegetables, and fruits; low sugar content distributed throughout the day; excluded whole grains, soft drinks, and lactose; match WCRF cancer prevention recommendations such as avoiding alcohol consumption	NA	18 months	CTCAE, daily diary	Patients with medium–high adherence to MMHD had a significantly lower risk of grade 2–3 diarrhea than those with low adherence (OR:0.5, *p* < 0.001), a higher number of chemotherapy cycles were associated with increased risk of grade 2–3 diarrhea (OR: 1.2, *p* = 0.02)	Adhering to a MMHD can be useful in controlling the severity of chemotherapy-induced diarrhea
Bauersfeld et al., 2018	STF: 60 h during either the first (c1-c3) or last half (c4-c6) of scheduled chemotherapy (36 h before to 24 h after chemotherapy) with normocaloric nutrition during the opposing half; only allowed water, tea, small amounts of vegetable juice, and light vegetable broth with a maximum daily calorie intake of 350 kcal; all participants received individual 1 h counseling by dietitians according to needs	Normocaloric Mediterranean diet period	4–6 chemotherapy cycles	Two interviews during and at the end of fasting, patient diary	Statistically significant beneficial effect (increase in QOL and reduction of fatigue with* p* = 0.041 and *p* = 0.006 respectively) of STF during c1-c3 in group A compared to STF during cycles c4-c6 in group B, the majority of patients reported better tolerance to chemotherapy with STF, 31 patients reported they would fast again during chemotherapy, no significant changes in weight, no grade III or IV toxicities reported	STF during the beginning of chemotherapy may lead to a better tolerance of chemotherapy and reduced fatigue
de Groot et al., 2020	FMD: 96 h or 4-day (3 days before chemotherapy and day 1 of cycle) plant-based, low amino-acid substitution diet consisting of soups, broths, liquids, and tea; allotted calories decreased from day 1 (~ 1200 kcal) to days 2–4 (~ 200 kcal); carbohydrates/proteins/fats energy ratio was approximately 3.5/1/2 on the first day and complex carbohydrates were the main macronutrient (> 80 energy%) on the subsequent 3 days	Habitual diet	8 cycles neo-adjuvant AC-T chemotherapy or 6 cycles neo-adjuvant FEC-T chemotherapy	CTCAE	Significantly less DNA damage in T-lymphocytes in the FMD group compared to the control (*p* = 0.045), radiologically complete or partial response occurred 3 times more often in the FMD group compared to the control (OR: 3.168, *p* = 0.039), 90–100% tumor cell loss is more likely when following a FMD (OR: 4.109, *p* = 0.016), no significant difference in grade III/IV toxicity or QOL between the two groups	FMD may limit the need for dexamethasone in protecting against chemotherapy toxicities, protect cells against DNA damage, and support better radiological outcomes
de Groot et al., 2015	STF: 48 h (24 h before to 24 h after start of chemotherapy) only allowed water, coffee, or tea without sugar	Healthy nutrition with a minimum of 2 pieces of fruit per day	6 cycles	CTCAE	y-H2AX levels in CD45 + CD3 + lymphocytes remained elevated in the control (*p* = 0.015) but not in the STF group after 7 days, y-H2AX levels in CD45 + CD3—myeloid cells were increased after 30 min in the control (*p* = 0.009) but not in the STF group, significantly higher erythrocyte (*p* = 0.007) and thrombocyte (*p* = 0.00007) cell counts after chemotherapy in the STF group compared to the control at day 7, no significant difference for leukocytes and neutrophils between the two groups, no significant difference in grade III/IV toxicity between the two groups, no grade V toxicity reported	STF may reduce chemotherapy-induced DNA damage, promote the recovery of DNA damage in healthy cells, and protect against hematological toxicity
Khodabakhshi et al., 2020	KD: 6% of calories from carbohydrates, 19% from protein, 20% from MCT oil, and 55% from fat; excluded grains, grain products, starchy vegetables, fruit, and sugar; mainly obtained carbohydrates from non-starchy vegetables, but a small amount of berries and nuts were allowed; obtained protein from egg, meat, poultry, and fish; weekly nutrition counseling session	Standard diet: 55% carbohydrates, 15% protein, and 30% fat	12 weeks	Not specifically reported but included the symptom subscale of EORTC QLQ-C30	At 6 weeks the KD group had better global QOL and improved physical activity compared to the control (*p* = 0.02, 0.01 respectively), diarrhea increased in the control at 6 weeks compared to the KD group (*p* = 0.02), lactate and ALP significantly decreased in the KD group compared to the control (*p* = 0.02, 0.007 respectively), no significant difference in QOL or physical activity between the two groups at 12 weeks; Within-group results: KD group had a within-group decrease in hunger at 12 weeks (*p* = 0.02); both groups had a within-group decrease in physical performance at 12 weeks, but this was only significant in the KD group (*p* = 0.04); within-group decrease in role and social functioning scores in the control group (*p* = 0.02), but not in the KD group	A KD may provide initial benefits in reducing diarrhea and improving physical activity; however, these benefits may not be sustainable
Kleckner et al., 2022	MedDiet: 4 weeks of weekly food provision compliant with MedDiet (12 frozen meals per week) then participants used a MedDiet cookbook provided to cook for the remaining 4 weeks, alcohol was not encouraged, educational sessions and handouts at week 3, weekly phone calls for all participants	Habitual diet	8 weeks	CTCAE, BFI, Symptom Inventory	Small-moderate improvement of fatigue in the MedDiet group compared to the control at weeks 4 and 8 (ES = 0.31, 0.25 respectively); significant association between Mediterranean diet adherence and FACIT-F scores (*p* < 0.05), BFI scores (*p* < 0.05), and symptom interference with QOL on the Symptom Inventory (*p* = 0.041); moderate-large improvement in fatigue for patients who had a low MedDiet score at baseline compared to those who had a higher score at baseline (ES = 0.67, 0.48 at week 4 and 8 respectively); statistically significant association between worse fatigue and lower overall mitochondrial function (lower basal respiration, maximal respiratory, and spare capacity)(*p* < 0.05); no significant change in fatigue over time for either group	MedDiet may have beneficial effects on cancer-related fatigue
Lugtenberg et al., 2021	FMD: 96 h or 4-day (3 days before chemotherapy and day 1 of cycle) plant-based, low amino-acid substitution diet consisting of soups, broths, liquids, and tea; allotted calories decreased from day 1 (~ 1200 kcal) to days 2–4 (~ 200 kcal); carbohydrates/proteins/fats energy ratio was approximately 3.5/1/2 on the first day and complex carbohydrates were the main macronutrient (> 80 energy%) on the subsequent 3 days	Habitual diet	8 cycles neo-adjuvant AC-T chemotherapy or 6 cycles neo-adjuvant FEC-T chemotherapy	Not specifically reported but included the symptom subscale of EORTC QLQ-C30 and QLQ-BR23	Statistically significant decrease in global health and physical, role, cognitive, and social function in both groups over treatment (*p* < 0.01); significant improvement in emotional functioning in both groups over treatment (*p* < 0.05); significant worsening of fatigue, pain, dyspnea, loss of appetitie, and constipation over the course of treatment in both groups (*p* < 0.01); FMD group had better scores in insomnia compared to the control, and the nausea the FMD group reported was not clinically relevant unlike the control; patients who were adherent to the FMD had better scores in all five functional scales and less reports of fatigue, nausea, and insomnia than those who were not adherent or part of the control (*p* < 0.05); FMD group had a decrease in BMI over the course of treatment but after a 6 month follow up their BMI did not differ, whereas the control had an increase in BMI over the course of treatment and at 6 month follow up (*p* = 0.026); FMD group had a significantly better understanding and less concern for side effects compared to the control (*p* < 0.05)	FMD may protect against common weight gain during breast cancer treatment. Those who are more adherent to a FMD diet may experience fewer chemotherapy toxicities than those who are not adherent
Omar et al., 2022	IF: 3 consecutive days (day before, during, and after chemotherapy) fasting 18 h from 12 am to 6 pm with eating for 6 h from 6 pm to 12 am; allowed water and small quantities of food consisting mainly of vegetables, fruits, and a small amount of proteins and carbohydrates with a limitation of sugar and fats; total quantity of calories consumed per day during 6 h of eating < 750 kcal	Balanced diet: lots of fruits and vegetables; moderate amounts of whole grains; plant protein sources such as nuts, beans, and lentils; small servings of fish, poultry, and lean meats; and nonfat or low-fat dairy foods	4 cycles	CTCAE	No reported experiences of mouth sores or diarrhea in the IF compared to 33.3% and 50% respectively in the control (*p* = 0.004 and *p* < 0.001 respectively); 8.3% and 70.8% of the IF group experienced vomiting and nausea respectively compared to 79.2% and 100% experienced vomiting and nausea respectively in the control (*p* < 0.001 and *p* = 0.009 respectively); no reports of constipation in the control compared to 33.3% experienced constipation in the IF group (*p* = 0.004); no significant difference in thrombocytosis, neutropenia, headache, hair loss, fatigue, or hematologic parameters between the two groups; no grade 4 or 5 toxicities reported	IF may reduce the incidence of chemotherapy toxicities such as nausea, vomiting, mouth sores, and diarrhea
Riedinger et al., 2020	STF: abstain from all non-caloric nutrition 24 h before to 24 h following each chemotherapy cycle; allowed to maintain adequate hydration with water, black coffee, or tea without sweetener	Balanced, normocaloric diet	At least 6 cycles	CTCAE	Significant within-group differences in mean QOL scores in the STF group (*p* = 0.015) but not in the control; fewer hospitalizations, improved hematologic parameters, and reduced instances of grade 2 or higher toxicities, along with fewer dose reductions or delays in the STF group compared to the control; no significant difference in mean QOL or treatment outcomes between the two groups; no significant weight loss from fasting	STF may reduce chemotherapy toxicities, hospitalizations, and the need for dose reductions or delay over the course of treatment
Safdie et al., 2009	STF: voluntarily fasted 48–140 h prior to and/or 5–56 h following chemotherapy; one participant also utilized a low glycemic diet for 24 h before fasting; another participant also utilized a liquid diet for 24 h after fasting	Habitual diet	Average of 4 cycles	CTCAE	Significantly reduced fatigue (*p* < 0.001) and weakness (*p* < 0.00193) and virtually absent vomiting and diarrhea reported in STF participants; only minor complaints of dizziness, hunger, and headaches during fasting, but these did not interfere with daily activities; any weight lost during fasting was rapidly recovered in most patients and did not lead to any harm; fewer and less severe chemotherapy toxicities with virtually absent nausea, vomiting, diarrhea, abdominal cramps, and mucositis reported by all 10 participants while at least one of these toxicities was reported in 5/6 participants who ate habitually at some point during their cycles; lower severity of toxicities reported by all 4 of the participants who fasted their whole treatment; no toxicities were reported to be increased during STF	STF may reduce or eliminate chemotherapy toxicities
Sathiaraj et al., 2023	Plant-based, high-protein: 1.2 g/kg body weight/day protein; macronutrient distribution of 25–30% fats, 55–60% carbohydrates, 10–15% proteins; whey supplement once daily (350 kJ and 10 g protein); intake of at least 5 servings of fruit and vegetables, low-fat dairy, locally available millets and whole grains; limited number of high calories fried foods and sweetened carbonated beverages; restricted all types of meat (poultry, fish, red meat, game, organ meats) but included eggs; weekly consultations with an oncology nutritionist	Habitual diet	12 weeks	FSI, HGS	Significant decrease in fatigue over time in the intervention group compared to the control (from 4.2 ± 1.94 to 2.47 ± 1.31, *p* < 0.001); statistically significant decrease in BMI (*p* < 0.015) and fat mass (*p* < 0.0001) in the intervention group compared to the control; significant improvement in percentage muscle mass (*p* < 0.01) in the intervention group and significant decrease in muscle mass in the control group (*p* < 0.05); handgrip strength improved in the intervention group while strength decreased in the control; fatigue scores were inversely associated with changes in muscle mass in both the intervention group (Pearson r = −0.09) and the control group (Pearson r = −0.041); greater intake of protein and fiber and reductions in total energy consumed in the intervention group compared to the control (*p* < 0.001)	Plant-based, high-protein diet may decrease fatigue, BMI, and fat mass while improving muscle mass percentage. The increase in muscle mass percentage may explain the reduction in fatigue
Zhang et al., 2023	Anti-inflammatory: ate personalized hospital food when hospitalized; ate according to educational manual when out-of-hospital and intake was monitored via photos of meals; 2 servings of fruits per day; 5 servings of vegetables per day; 3 servings of whole grain cereals per day; 3 or more servings of fish per week; 2 servings of nuts and seeds per day; 2 servings of olive oil or linseed oil per day; 2 servings of probiotic low-fat dairy products per day; no more than 2 servings of red meat per week; 5 servings of white meat per week; recommends ginger, green onion, cinnamon, turmeric, and white tea or green tea; does not recommend refined carbohydrates, trans-fatty acids, sugar, or processed meats and soft drinks; all participants underwent 4 individual nutritional consultations	Usual nutrition recommendations based on dietary nutrition prescriptions for cancer patients in China	3 months	Chinese version BFI	Significant improvement in fatigue in the intervention group before the 4th admission assessment, whereas the control had increasing fatigue over time (*p* < 0.001); hs-CRP levels were significantly lower in the intervention group compared to the control before the 3rd chemotherapy cycle (*p* < 0.001); intervention group had a significantly greater improvement in emotional function (*p* = 0.02), cognitive function (*p* = 0.02), fatigue (*p* = 0.01), sleep disturbances (*p* = 0.02), and constipation (*p* = 0.01) compared to the control; no significant difference in improvement of fatigue from baseline to prior to the 3rd chemotherapy cycle between the two groups; intervention resulted in no reported adverse events	Anti-inflammatory diet may reduce inflammation and fatigue over time
Zorn et al., 2020	mSTF: 4-day (96 h) period: 3 days before and 1 day following chemotherapy; starting and ending at 6 pm, where the caloric restriction was limited to 25% of patient's energy requirement determined by nutritionist (average kcals consumed was 400–600 kcal/day); individually adapted, very low-calorie diet with a ketogenic macronutrient composition (75% fats, 15% protein, 10% carbohydrates); minimum 2.5 L of calorie-free liquid including water, herbal tea, and diet drinks without caffeine or alcohol; 6-day normocaloric KD diet prior to mSTF: received advice and recipes to follow KD according to own food preferences; limit carbohydrates to 20–40 g/day; 75% fats, 15% protein, 10% carbohydrates; no limit on caloric content; Group A: mSTF / NC, Group B: NC / mSTF, Group C: KD + mSTF / NC, Group D: NC / KD + mSTF	Normocaloric diet (NC)	2 periods of 2–3 cycles with one period being either mSTF or KD + mSTF and one period being NC	CTCAE, self-report questionnaire	Significantly less frequent stomatitis (*p* = 0.013), fewer and less severe headaches (*p* = 0.002), less frequency and severity of weakness (*p* = 0.0024), and fewer, less severe overall toxicities in the mSTF group compared to the control (*p* = 0.023); significantly fewer chemotherapy postponements in the mSTF groups compared to the control (*p* = 0.034); significant fat loss leading to significant weight loss that remained at the end of the study in the mSTF groups compared to the control (*p* < 0.005), however, lean body mass remained stable in both groups; no significant difference in N/V/D, leucocytes, thrombocytes, neutrophils, urea, creatinine, liver parameters, and CRP between the mSTF groups and the control; no significant differences in fatigue, CIPN, or QOL between the mSTF groups and the control; no significant differences in any aspect between the mSTF alone and the mSTF in combination with a KD; the most common fasting-related side effects were hunger, dizziness, weakness, and headaches	mSTF may reduce chemotherapy toxicities, improve chemotherapy tolerance, and result in faster physical regeneration after chemotherapy. There are no beneficial effects to implementing a 6-day KD prior to a mSTF

*STF*, short-term fasting; *FMD*, fasting-mimicking diet; *KD*, ketogenic diet; *MedDiet*, Mediterranean Diet; *IF*, intermittent fasting; *mSTF*, modified short-term fasting; *NA*, not applicable; *QOL*, quality of life; *CTCAE*, common terminology criteria for adverse events; *OR*, odds ratio; *c1-c4*, chemotherapy cycle 1–4; *WCRF*, World Cancer Research Fund; *FACIT*, functional assessment of chronic illness therapy; *AC-T*, doxorubicin/cyclophosphamide followed by docetaxel; *FEC-T*, fluorouracil/epirubicin/cyclophosphamide; *EORTC-QLQ-C30 (BR23)*, European Organization for Research and Treatment of Cancer Core Quality of Life questionnaire (breast cancer specific module); *BFI*, brief fatigue inventory; *DNA*, deoxyribonucleic acid; *y-H2AX*, protein that depicts DNA damage; *CD45/3*, proteins; *MCT oil*, medium-chain triglycerides; *ALP*, alkaline phosphatase; *BMI*, body mass index; *FSI*, fatigue symptom inventory; *HGS*, hand grip strength; *NC*, normocaloric; *N/V/D*, nausea/vomiting/diarrhea; *CRP*, c-reactive protein

## Results

### Study characteristics

A majority of the articles (11/14, 78.6%) were published in or after 2020 [10, 16–25]. Three articles were published prior to 2020 (including one in 2009 [26], 2015 [27], 2018 [28]). Details on study characteristics, participants, diet interventions, outcome measures, results, and quality are provided in Tables 1 and 2. Ten of the 14 studies (71.4%) in this review were RCTs [16, 17, 19–22, 24, 25, 27, 28], 2 (14.3%) were quasi-experimental [10, 23], 1 (7.1%) was a case series [26], and 1 was a cohort study [18]. Two studies utilized a cross-over design [23, 28]. Of the 10 RCTs, 2 used data from the DIRECT trial [16, 17], and 4 were pilot trials [23, 24, 27, 28]. Common limitations of the included articles were small sample sizes, heterogeneity in the study populations, single-center studies, and other potential biases (selection and self-report bias). Study-specific limitations are presented in Table 1.

The sample size of the studies ranged from 10 to 137 participants. Eight articles (57.1%) included only female participants, and these studies focused specifically on breast and gynecologic malignancies [16, 17, 19, 21, 23, 25, 27, 28]. Of the 14 studies, 6 included solely breast malignancies [16, 17, 19–21, 27]; 2 included colorectal malignancies [10, 18]; 1 included lung malignancies [22]; 1 included breast and ovarian malignancies [28]; 1 included ovarian, uterine, or cervical malignancies [25]; and 1 included all gynecologic malignancies [23]. Two studies included participants with any malignancy [24, 26]. Even though one RCT did not restrict recruitment based on specific malignancies, it ended up including a majority of participants (90.9%) with breast cancer [24]. Additionally, 93.9% of the participants in this study were female [24]. Cancer staging varied from stage I to stage IV across the studies, and the articles included a range of chemotherapy regimens. These details are presented in Table 1.

This review highlights articles that address a range of dietary patterns. Eight of the 14 studies tested a variation of fasting: 4 studies a short-term fast (STF) [25–28], 2 an intermittent fast (IF) [18, 20], and 2 a fasting-mimicking diet (FMD) [16, 17]. Besides fasting, 2 studies tested a Mediterranean diet [10, 24]; 1 tested a ketogenic diet [19]; 1 tested a plant-based, high-protein diet [21]; and 1 tested an anti-inflammatory diet [22]. One study simultaneously investigated short-term fasting and a ketogenic diet [23]. Study durations varied and are presented in Table 2.

### Toxicity assessment methods

The studies included in this review (N = 14) all measured chemotherapy toxicities. The most common measurement tool, utilized in eight of the articles, was the National Cancer Institute’s Common Terminology Criteria for Adverse Events (CTCAE) [10, 16, 20, 23–27]. Two articles utilized patient questionaries to document toxicities [18, 23], 2 utilized patient diaries [10, 28], and 1 utilized interviews [28]. Furthermore, two studies analyzed toxicity through the symptom subscales of the European Organization for Research and Treatment of Cancer Quality of Life Questionnaire and breast cancer-specific module (EORTC QLQ-C30 and QLQ-BR23) [17, 19].

Fatigue was specifically measured in 3 studies [21, 22, 24]. Two studies measured fatigue through the Brief Fatigue Inventory (BFI) [22, 24]. One study also measured fatigue, sleep problems, and drowsiness on a Symptom Inventory scale rated similarly to BFI [24]. Another study measured fatigue with the Fatigue Symptom Inventory (FSI) and handgrip strength (HGS) [21].

### Level of evidence

Melnyk and Fineout-Overholt’s hierarchy of evidence for intervention questions was utilized in this review [29]. Ten of the 14 articles in this review are RCTs and were graded as “Level 2” [16, 17, 19–22, 24, 25, 27, 28]. This is the strongest research design to determine the relationship between diet and chemotherapy toxicities. Two of the 14 studies were graded as “Level 3” [10, 23]. One study was graded as “Level 5” [18] and one was graded as “Level 6” [26].

### Quality appraisal

Supplementary Tables 1 to 4 present the results of JBI’s critical appraisals for the included studies. The ratings ranged from fair to excellent. Five articles (35.7%) were given an excellent-quality rating [16, 17, 20, 22, 23]. Eight studies (57.1%) were given a good-quality rating [18, 19, 21, 24–28]. One study was rated as having fair quality [10]. Among the good quality RCTs, the most common issues were as follows: not blinding participants [16, 17, 19–22, 24, 25, 27, 28], those delivering the treatment [17, 19–22, 24, 25, 27, 28], or outcomes assessors [17, 19, 20, 22, 24, 25, 27, 28]; treatment groups not receiving identical care outside of the diet (e.g. nutrition counseling in the intervention group but not in the control) [19, 21, 24]; and not analyzing participants in the groups to which they were randomized [21, 25, 28]. One RCT [27] failed to conceal allocation to treatment groups and one [21] failed to report on follow-up attrition. Among the quasi-experimental studies, the article assessed as having good quality lacked comparable participants between the groups, and the different groups did not receive similar care aside from the exposure (intervention) [23]. The other quasi-experimental study was assessed as having fair quality due to the absence of randomization and a control or comparison group [10]. The cohort study was assessed as having good quality; however, it failed to report on follow-up attrition and action taken to deal with confounding variables [18]. The case series was assessed as having good quality; however, this article failed to provide sampling methods (such as complete inclusion of participants) [26].

#### Narrative synthesis

There were 5 categories of dietary interventions employed in the 14 articles included in this review: variations of fasting; a Mediterranean diet; a ketogenic diet; a plant-based, high-protein diet; and an anti-inflammatory diet. All the dietary patterns implemented in the studies were suggested to be feasible, safe, and generally accepted by study participants. Additional information about the studies’ interventions, controls, and participants is presented in Tables 1 and 2.

### Dietary Intervention 1: Fasting

The studies that investigated fasting were conducted in the following countries: Saudi Arabia [18], Germany [23, 28], Netherlands [16, 17, 27], Egypt [20], and the United States [25, 26]. Among the 9 studies evaluating fasting, there was significant heterogeneity between the interventions. Studies implemented a short-term fast (STF) [23, 25–28], an intermittent fast (IF) [18, 20], and a fasting-mimicking diet (FMD) [16, 17]. Even within the specific fasting protocols, there were variations in fasting duration (18 h to 96 h), study duration (2 to 8 chemotherapy cycles), caloric intake (abstain from all caloric nutrition to a daily intake of less than 1200 kcals), and the types of foods allowed (refrain from eating and drinking altogether to small quantities of fruits and vegetables). Details on each study’s intervention are presented in Table 2.

Various articles found fasting to be beneficial in reducing the severity and frequency of chemotherapy toxicities [16, 17, 20, 23, 25–28]. The benefits include reduced (1) mouth sores [20, 23, 26], (2) diarrhea [20, 26], (3) vomiting and nausea [17, 20, 26], (4) abdominal cramps [26], (5) long-term weight gain [17], (6) insomnia [17], (7) headaches [23], (8) weakness [23, 26], and (9) fatigue [26]. Additionally, one author found a reduced need for dexamethasone before chemotherapy [16] and significantly higher erythrocyte and thrombocyte cell counts that may represent less severe bone marrow suppression or decreased cell breakdown while fasting [27]. Furthermore, two studies reported fasting may reduce DNA damage during chemotherapy [16, 27]. Participants who fasted saw lower y-H2AX levels, the protein formed when a DNA strand breaks, in CD45 + CD3 + lymphocytes than those who did not fast [16, 27].

Fasting interventions were also shown to contribute to a better treatment experience in patients currently receiving chemotherapy [17, 25]. One article revealed that patients following a FMD felt as if their cancer treatment had less of an impact on their lives and were less concerned about the toxicities of chemotherapy in comparison to those who followed their habitual diet [17]. Furthermore, four articles suggest that following a fasting intervention during chemotherapy may improve patients' ability to tolerate their treatment regimen [18, 23, 25, 28]. Two studies reported a majority of participants had improved tolerability to chemotherapy when fasting [18, 28]. Another two studies found fewer chemotherapy postponements or dose reductions in those who fasted [23, 25]. One of these studies also reported lower unanticipated hospitalizations in the fasting group compared to the control [25].

Adherence may play a role in the effect fasting has on chemotherapy toxicities [17, 18, 26]. Two studies reported participants who were more adherent to the fasting intervention had reduced severity and frequency of chemotherapy toxicities than those who were not adherent [17, 26]. Moreover, one study found that fasting may be more effective if implemented at the start of chemotherapy rather than waiting till the middle of treatment to begin fasting; participants who began fasting at the start of chemotherapy experienced greater reductions in fatigue [28].

### Dietary Intervention 2: Mediterranean Diet

The studies that investigated a Mediterranean Diet were conducted in Scotland [10] and the United States [24]. One study implemented a Mediterranean Modified Healthy Diet (MMHD) for 18 months that restricted caloric intake to 1818 kcals, limited various foods and food groups, and avoided alcohol (Table 2) [10]. This article showed reduced diarrhea in those more adherent to the dietary pattern. The other study provided participants with frozen meals adherent to a Mediterranean Diet for 4 weeks and then had participants follow a Mediterranean Diet cookbook for another 4 weeks. Alcohol was not encouraged [24]. At the end of this study, this dietary pattern was shown to reduce fatigue, with an even greater effect observed in those who had a low Mediterranean score at baseline. Furthermore, there was a significant association between Mediterranean diet adherence and FACIT-F scores, BFI scores, and symptom interference with QOL on the Symptom Inventory [24].

### Dietary Intervention 3: Ketogenic Diet

The studies that investigated a ketogenic diet were conducted in Germany [23] and Iran [19]. One study implemented a 6-day normocaloric ketogenic diet over 2–3 chemotherapy cycles, with 75% of calories coming from fat while carbohydrate intake was limited to 20–40 g per day [23]. The other study implemented a 12-week ketogenic diet, with 6% of calories coming from carbohydrates and 55% coming from fat (Table 2) [19]. The studies reported mixed outcomes. One study reported short-term outcomes [19]. The ketogenic diet was shown to provide initial improvements in diarrhea incidence and physical activity; however, these benefits were not sustainable throughout treatment. In fact, there was a significant decline in physical performance from baseline in those following the dietary pattern [19]. The other article did not find any added benefits from the ketogenic diet when combined with fasting [23].

### Dietary Intervention 4: Plant-Based, High-Protein Diet

The study that investigated a plant-based, high-protein diet was conducted in India [21]. The dietary pattern (Table 2) was shown to reduce fatigue during chemotherapy, possibly through an increase in muscle mass. The intervention group showed a significant decrease in average fatigue over time and a significant increase in muscle mass, whereas the habitual diet group did not experience any reduction in fatigue and showed a decrease in muscle mass over time. Muscle mass was negatively correlated with fatigue in both the intervention group and the control group.

### Dietary Intervention 5: Anti-Inflammatory Diet

The study that investigated an anti-inflammatory diet was conducted in China [22]. The dietary pattern (Table 2) was shown to reduce fatigue, possibly through a decrease in inflammation. After 3 months, the intervention group showed significantly lower hs-CRP levels, a protein that increases when there is inflammation present, compared to the control. There was a significant reduction in fatigue in the anti-inflammatory diet group before the 4th cycle compared to those following usual nutrition.

## Discussion

To our knowledge, this is the first systematic review on alleviating chemotherapy toxicities that does not limit the analysis to a specific dietary intervention and specific type of quantitative design. This review identified quantitative literature (N = 14) testing a variety of dietary patterns including fasting; a ketogenic diet; a Mediterranean diet; a plant-based diet, high-protein diet; and an anti-inflammatory diet to enhance patients’ experience with chemotherapy. While the results are not definitive, it is suggested that dietary modification during chemotherapy treatment may benefit patients by reducing toxicities, such as fatigue, and improving tolerability.

The majority of articles included in this review investigate fasting interventions. In rodent studies, fasting has been shown to increase the sensitivity of cancer cells to chemotherapy while protecting healthy cells against its toxicities, highlighting the need for human trials [17, 30]. Research has supported the feasibility and safety of fasting in human groups receiving chemotherapy. Chronic fasting or caloric loss is not suitable for patients with cancer due to its risk of unintended weight loss, however, shorter durations of fasting do not pose this risk. Furthermore, it is important to consider individuals’ nutritional status when considering fasting and confine the intervention to well-nourished patients to prevent adverse health effects from weight loss [31, 32]. The articles in this review explore STF, IF, and FMD. The analysis did not differentiate between the variations of fasting, as there was no clear distinction between them regarding hours fasted and how much caloric consumption was allowed during the fasting period.

The benefits of fasting likely stem from the suppression of gastrointestinal function. The suppression can trigger systemic metabolic changes that may improve treatment outcomes [33]. The phenomenon of differential stress resistance (DSR) may explain how fasting can help patients avoid toxicities from chemotherapy. DSR implies that fasting shields healthy cells, which become inactive and resistant due to nutrient scarcity, from chemotherapy while cancer cells, which focus on growth over repair, remain vulnerable to chemotherapy’s effects and toxicities [34]. The results of this systematic review support DSR, showing that patients who fasted generally experienced fewer or less severe toxicities [17, 20, 23, 25–27], were protected from DNA damage [16, 27], tolerated chemotherapy better [18, 23, 25, 28], and did not require dexamethasone for toxicity protection [16]. More evidence is needed to confirm these suggestions.

The Mediterranean diet; plant-based, high-protein diet; and anti-inflammatory diet also showed promising results in supporting patients through chemotherapy treatment. The Mediterranean diet is a non-restrictive eating plan rich in fruits, vegetables, legumes, nuts, whole grains, and olive oil, and low in red meat and processed foods. It addresses nutrient deficiencies, supports a healthy gut microbiome, and aids in the metabolism of protein, fiber, polyphenols, and hydroxytyrosol [35–37]. This dietary pattern may reduce chemotherapy toxicities by lowering inflammation, oxidative stress, and metabolic dysfunction [35–37]. Additionally, its proven ability to reduce fatigue in chronic conditions like multiple sclerosis suggests similar benefits for cancer patients [37]. The results of this systematic review support this hypothesis. It was suggested that a Mediterranean diet may help reduce diarrhea [10] and fatigue [24] in patients undergoing chemotherapy, and its effectiveness could be influenced by how well patients adhere to the dietary pattern [24]. Furthermore, the diet may have a greater impact on patients who were not already following a Mediterranean diet before starting chemotherapy [10].

Likewise to a Mediterranean diet, an anti-inflammatory diet is also thought to reduce chemotherapy toxicities due to its ability to reduce inflammation. An anti-inflammatory diet combines multiple anti-inflammatory foods such as fruits, vegetables, fiber, and n-3 polyunsaturated fatty acids while eliminating pro-inflammatory foods such as red meat, saturated fatty acids, refined grains, and simple carbohydrates; this eating plan may attenuate the inflammatory effects of chemotherapy that cause cell and tissue damage [22, 38, 39]. The review findings indicate an anti-inflammatory diet may improve fatigue through decreased inflammation. It was also suggested that the benefits of this dietary pattern may take time to become apparent [22].

Furthermore, a plant-based, high-protein diet may help mitigate the toxic toxicities of chemotherapy due to its anti-inflammatory, antioxidant, and muscle-building benefits. This dietary pattern primarily includes fruits, vegetables, and protein-rich sources such as legumes and lentils [21]. Additionally, adequate protein intake has been linked to reduced cancer-related fatigue, a frequent chemotherapy toxicity [40]. Protein intake is also crucial for maintaining or building muscle mass. The findings of this paper support the idea that a plant-based, high-protein diet may effectively combat fatigue by promoting muscle growth in patients undergoing chemotherapy [21].

The ketogenic diet did not show as promising results as the other interventions in this review paper. Although there are variations in the levels of carbohydrate restrictions and fat levels, a classic ketogenic diet typically consists of 90% fat, 2% carbohydrates, and 8% protein [41]. The ketogenic diet, by mimicking starvation and inducing ketosis, may offer anti-inflammatory and pro-apoptotic effects [42]. Despite the growing evidence that a ketogenic diet has the potential to alleviate chemotherapy toxicities, there are concerns about this dietary pattern having a negative impact on cancer patients’ well-being [42, 43]. Following this restrictive eating plan long-term may lead to micronutrient deficiencies, appetite reduction, nausea, constipation, fatigue, hyperlipidemia, and unintended weight loss [19]. This review suggests that while the ketogenic diet may provide some initial benefits, these benefits may not be sustainable, and the diet may be associated with a decline in physical performance over time [19].

### Strengths and limitations

A key strength of this systematic review is its broad scope, as it did not restrict studies to a specific quantitative design, malignancy type, dietary intervention, or chemotherapy regimen. The heterogeneity among the articles enhances the generalizability of the findings and allows for a more comprehensive understanding, bringing us closer to formulating dietary recommendations for cancer patients undergoing chemotherapy. Additionally, an extensive literature search was conducted across multiple databases to ensure that all relevant studies meeting the criteria were included. Notably, the majority of the articles (11/14) were published within the last four years, reflecting recent advancements in the field.

This review is not without limitations. The variations in both dietary interventions and cancer types or stages may have introduced inconsistencies, making it more challenging to identify clear patterns or draw firm conclusions. Additionally, other factors may have influenced the relationship between dietary patterns and chemotherapy toxicities. We did not specifically evaluate other medications aside from chemotherapy; however, 5/14 studies reported that patients also received biological therapies [18] or other pharmacological treatments, such as antiemetics or dexamethasone [16, 20, 27, 28]. While the studies examined various malignancies, the majority (9/14) focused specifically on female breast and gynecological cancers, which limits the generalizability of the findings to other cancer types. Furthermore, only one or two articles investigated each of the ketogenic; Mediterranean; plant-based, high-protein; and anti-inflammatory diets, providing insufficient evidence to make definitive conclusions about the effectiveness of these dietary patterns in reducing chemotherapy toxicities.

### Implications for future research

Addressing the limitations outlined in this review is critical to advancing the field. Future research must focus on identifying effective dietary interventions that alleviate chemotherapy toxicities across all cancer patients. Additionally, research should be expanded to include various cancers such as hematological malignancies. Rigorous, well-designed, multi-centered studies with larger sample sizes are essential to evaluate different dietary patterns and establish concrete evidence. A large-scale, longitudinal study would be particularly valuable for assessing the true impact of diet on chemotherapy toxicities in both the short and long term.

## Conclusions

The results of this systematic review do not offer conclusive evidence to determine which dietary pattern is most advantageous in reducing chemotherapy toxicities in patients currently receiving treatment. Fasting interventions showed diverse results, with studies demonstrating varying levels of improvements in chemotherapy toxicities. Particularly, fatigue may be alleviated by fasting; a Mediterranean diet; a plant-based, high-protein diet; or an anti-inflammatory diet. The ketogenic diet might offer initial advantages in improving diarrhea and physical activity; however, these improvements may not be long-lasting, and physical performance could deteriorate over time. All the dietary interventions analyzed show the potential to help patients during chemotherapy. Evidently, additional research is required to provide more evidence to reach definitive conclusions about which dietary pattern(s) can be effectively implemented in clinical practice and improve patients’ treatment experiences and outcomes.

## Supplementary Information

Below is the link to the electronic supplementary material.

Supplementary file1 (PDF 136 KB)

## Data Availability

No datasets were generated or analysed during the current study.

## Appendix

PubMed

- (((((("Quality of Life"[Mesh] OR "life quality" OR toxicit*) OR ("Perception"[Mesh])) OR ("Drug-Related Side Effects and Adverse Reactions"[Mesh])) OR ("Drug Tolerance"[Mesh])) OR ("Patient Satisfaction"[Mesh])) OR ("Cancer Pain"[Mesh])) AND (("Diet"[Mesh]) OR ((((((("Diet, Ketogenic"[Mesh])) OR "Fasting"[Mesh]) OR "Diet, Western"[Mesh]) OR "Diet, Mediterranean"[Mesh]) OR "Caloric Restriction"[Mesh]) OR "Diet, Vegetarian"[Mesh])) OR (alternative diet*) AND (chemotherap*) AND (("Neoplasms"[Mesh]) OR (malignan*) OR cancer*)

CINAHL

- ((MH "Neoplasms + ") OR malignan* OR cancer*) AND ((MH "Diet + ") OR (MH "Fasting + ") OR (MH "Diet, Western") OR (MH "Diet, Ketogenic") OR (MH "Mediterranean Diet") OR "caloric restriction" OR (MH "Plant-Based Diet") OR "alternative diet*") AND (chemotherap*) AND ((MH "Quality of Life + ") OR “life quality” OR (MH "Drug Toxicity + ") OR (MH "Perception + ") OR (MH "Adverse Drug Event + ") OR (MH "Drug Tolerance") OR (MH "Patient Satisfaction + ") OR (MH "Cancer Pain"))

Embase

- ('malignant neoplasm'/exp OR 'malignant neoplasm' OR cancer* OR malignant*) AND ('diet'/exp OR 'ketogenic diet'/exp OR 'fasting'/exp OR 'caloric restriction'/exp OR 'Mediterranean diet'/exp OR 'vegetarian diet'/exp OR 'Western diet'/exp OR ‘alternative diet*’) AND ('chemotherapy'/exp OR 'chemotherap*') AND ('quality of life'/exp OR 'life quality' OR 'toxicity'/exp OR 'perception'/exp OR 'adverse drug reaction'/exp OR 'drug tolerance'/exp OR 'patient satisfaction'/exp OR 'cancer pain'/exp)
