# Supplementary material for: Evaluating the role of dietary interventions in reducing chemotherapy toxicities in cancer patients: a systematic review
Source: J Cancer Surviv. 2025 Mar 22;20(Suppl 1):962–81. doi: 10.1007/s11764-025-01777-6 (PMC13676626; doi:10.1007/s11764-025-01777-6)
Supplement: Supplementary file 1 [file 11764_2025_1777_MOESM1_ESM.pdf]

Article Title: Evaluating the Role of Dietary Interventions in Reducing Chemotherapy Toxicities in Cancer Patients: A Systematic Review

Journal Name: Journal of Cancer Survivorship

Author Names: Jessica Abene BSN, RN [1*] and Jie Deng PhD, RN, OCN, FAAN [1]

Affiliation: [1]University of Pennsylvania, School of Nursing, 418 Curie Blvd, Philadelphia, PA

Correspondence: jabene@nursing.upenn.edu

Supplementary Table 1. Quality appraisal rating for included RCT.

Study	True randomization	Allocation concealment	Treatment groups similar at baseline	Participants blind	Those delivering treatment blind	Outcome assessors blind	Treatment groups treated identically other than intervention	Follow-up complete	Participants analyzed in groups to which randomized	Outcomes measured the same for all participants	Outcomes measured in reliable ways	Appropriate statistics	Appropriate design	Total out of 13 (%)
Bauersfeld et al., 2018	1	1	1	0	0	0	1	1	0	1	1	1	1	9 (69.2)
de Groot et al. 2020	1	1	1	0	1	1	1	1	1	1	1	1	1	12 (92.3)
de Groot et al. 2015	1	0	1	0	0	0	1	1	1	1	1	1	1	9 (69.2)
Khodabakhshi et al., 2020	1	1	1	0	0	0	0	1	1	1	1	1	1	9 (69.2)
Kleckner et al., 2022	1	1	1	0	0	0	0	1	1	1	1	1	1	9 (69.2)
Lugtenberg et al., 2021	1	1	1	0	0	0	1	1	1	1	1	1	1	10 (76.9)
Omar et al., 2022	1	1	1	0	0	0	1	1	1	1	1	1	1	10 (76.9)
Riedinger et al., 2020	1	1	1	0	0	0	1	1	0	1	1	1	1	9 (69.2)
Sathiaraj et al., 2023	1	1	1	0	0	1	0	0	0	1	1	1	1	8 (61.5)
Zhang et al., 2023	1	1	1	0	0	0	1	1	1	1	1	1	1	10 (76.9)

1, yes; 0, no/unclear; X, not applicable; 0-25%, poor; 26-50%, fair; 51-75%, good; 76-100%, excellent.

Supplementary Table 2. Quality appraisal rating for included quasi-experimental studies.

Study	Clear cause and effect	Control group present	Participants comparable between groups	Groups receiving similar care aside from exposure (intervention)	Outcome measured pre/post-intervention	Outcomes measured similarly in all groups	Reliable outcome measurements	Follow-up complete	Appropriate statistics	Total out of 9 (%)
Zorn et al., 2020	1	1	0	0	1	1	1	1	1	7 (77.8)
Artale et al., 2022	1	0	X	X	1	X	1	0	1	4 (44.4)

1, yes; 0, no/unclear; X, not applicable; 0-25%, poor; 26-50%, fair; 51-75%, good; 76-100%, excellent.

Supplementary Table 3. Quality appraisal rating for included case series.

Study	Clear inclusion criteria	Condition measurement reliable	Identification of condition valid	Consecutive sampling method	Complete inclusion of participants	Participant demographics reported	Clear reporting of participants' clinical information	Outcomes/follow-up reported	Site/clinic demographics reported	Appropriate statistics	Total out of 10 (%)
Safdie et al., 2009	0	1	1	0	0	1	1	1	X	1	6 (60)

1, yes; 0, no/unclear; X, not applicable; 0-25%, poor; 26-50%, fair; 51-75%, good; 76-100%, excellent.

Supplementary Table 4. Quality appraisal rating for included cohort studies.

Study	Two groups similar and recruited from the same population	Exposures measured similarly to assign participants	Valid and reliable exposure measurement	Confounding factors identified	Dealt with confounding factors	Participants free of outcome at study start	Valid and reliable outcome measurement	Appropriate follow-up time	Follow-up complete	Strategies to address incomplete follow-up	Appropriate statistics	Total out of 11 (%)
Alshammari et al., 2022	1	1	1	1	0	1	1	1	0	0	1	8 (72.7)

1, yes; 0, no/unclear; X, not applicable; 0-25%, poor; 26-50%, fair; 51-75%, good; 76-100%, excellent.
